# Inhibition of melanocortin 1 receptor slows melanoma growth, reduces tumor heterogeneity and increases survival

**DOI:** 10.18632/oncotarget.8372

**Published:** 2016-03-25

**Authors:** Rita G. Kansal, Matthew S. McCravy, Jacob H. Basham, Joshua A. Earl, Stacy L. McMurray, Chelsey J. Starner, Michael A. Whitt, Lorraine M. Albritton

**Affiliations:** ^1^ Department of Microbiology, Immunology and Biochemistry, College of Medicine, University of Tennessee Health Science Center, Memphis, TN 38163, USA

**Keywords:** melanoma, MC1R, agouti, B16-F10, GFP

## Abstract

Melanoma risk is increased in patients with mutations of melanocortin 1 receptor (MC1R) yet the basis for the increased risk remains unknown. Here we report in vivo evidence supporting a critical role for MC1R in regulating melanoma tumor growth and determining overall survival time. Inhibition of MC1R by its physiologically relevant competitive inhibitor, agouti signaling protein (ASIP), reduced melanin synthesis and morphological heterogeneity in murine B16-F10 melanoma cells. In the lungs of syngeneic C57BL/6 mice, mCherry-marked, ASIP-secreting lung tumors inhibited MC1R on neighboring tumors lacking ASIP in a dose dependent manner as evidenced by a proportional loss of pigment in tumors from mice injected with 1:1, 3:1 and 4:1 mixtures of parental B16-F10 to ASIP-expressing tumor cells. ASIP-expressing B16-F10 cells formed poorly pigmented tumors in vivo that correlated with a 20% longer median survival than those bearing parental B16-F10 tumors (p=0.0005). Mice injected with 1:1 mixtures also showed survival benefit (p=0.0054), whereas injection of a 4:1 mixture showed no significant difference in survival. The longer survival time of mice bearing ASIP-expressing tumors correlated with a significantly slower growth rate than parental B16-F10 tumors as judged by quantification of numbers of tumors and total tumor load (p=0.0325), as well as a more homogeneous size and morphology of ASIP-expressing lung tumors. We conclude that MC1R plays an important role in regulating melanoma growth and morphology. Persistent inhibition of MC1R provided a significant survival advantage resulting in part from slower tumor growth, establishing MC1R as a compelling new molecular target for metastatic melanoma.

## INTRODUCTION

Normal human and mouse melanocytes and melanoma cells express MC1R, a G-protein coupled receptor that regulates the biogenesis and maintenance of melanosomes, the specialized lysosomal compartments within which melanin pigments are synthesized. Ligation of MC1R by its primary agonist alpha-melanocyte stimulating hormone (α-MSH) up-regulates melanogenesis and induces expression of enzymes in the eumelanin biosynthetic pathways in human and mouse skin melanocytes and cutaneous melanoma cells. Pigment synthesis is also regulated by the natural inverse agonist of MC1R, human ASIP [1, 2] and its homolog mouse ASIP protein (agouti switch protein or agouti signaling protein) [3]. ASIP abates α-MSH-induced signaling by competing for the same binding site, and by inverse agonist action that modulates MC1R signaling in a way different from that induced by α-MSH [4-8]. α-MSH-induced signaling acts primarily through up-regulation of the microphthalmia-associated transcription factor (Mitf) [9], whereas, ASIP-induced signaling inhibits pigment synthesis by down-regulating translation of Tryp1 mRNA, an enzyme in the melanin synthesis pathways [10].

Mutations in MC1R are moderate risk factors for melanoma in humans that are thought to act by loss of function ([11-15] and reviewed in [16, 17]). Mutations in human ASIP are also moderate risk factors for melanoma, albeit ones with low penetrance [18, 19]. MC1R mutations associated with fair skin and red hair [20] may increase melanoma risk through insufficient pigment protection of genomic DNA from ultra-violet light damage; however, some MC1R mutations that increase melanoma risk are not associated with fair skin and red hair and do not alter pigment synthesis [21]. These observations indicate that a pigment-independent mechanism exists, but the basis for this increased risk has not yet been identified and it is not known which MC1R signaling pathway is involved in the pigment-independent mechanism, α-MSH agonist- or ASIP-inverse agonist induced signaling. In favor of loss of agonist induced MC1R signaling being involved in both pigment-dependent and independent mechanisms, it is known that α-MSH stimulates melanocyte precursor differentiation and inhibits melanocyte and melanoma cell migration in cell culture [22-24], and siRNA depletion of the downstream α-MSH effector, Mitf, increased B16 melanoma cell colonization in lungs [25], although coinjection of α-MSH and murine B16-F1 melanoma cells into mice did not reduce lung tumors [26]. While ASIP stimulates opposite effects, that is, de-differentiation of melanocytes, human melanoma and murine B16-F1 melanoma cells, and increases their migration in wound healing assays [24, 27], the effect of ASIP induced MC1R signaling on melanoma colonization *in vivo* is not known.

In addition, the possibility that MC1R is a growth receptor for melanoma has not been determined. While α-MSH stimulates melanocyte precursor proliferation *in vitro* suggesting that MC1R is a melanocyte precursor growth receptor, there is conflicting *in vitro* evidence on a role for MC1R as a melanoma growth receptor and this possibility has also not been examined *in vivo*. Purified recombinant ASIP (rASIP) inhibited growth of the B16-F1 sub-line at levels similar to growth inhibition by α-MSH, *e.g.*, 20-40% reductions [8, 10]. However, in three contrasting reports, a four-day exposure to rASIP or to a synthetic peptide derived from human ASIP (YY-ASIP) did not affect growth of melan-a melanocytes, B16-F1 cells, and attractin or mahogunin null melanocytes [4, 27, 28], whereas full-length ASIP inhibited proliferation [27]. Growth inhibition was not noted when ASIP was overexpressed in A2058 human melanoma cells *in vitro* [10].

Taking advantage of the lack of ASIP expression in the B16 melanoma and its sub-lines due to homozygous insertion of a transposable element in the first intron of the *a* gene encoding ASIP [29], we generated B16-F10 cells stably expressing an ASIP cDNA and compared their colonization, tumor growth and survival outcomes when implanted in syngeneic C57BL/6 mice to that of the parent ASIP-negative B16-F10 cells to investigate a possible role of MC1R in regulating tumor colonization and growth that could be involved in the melanoma risk associated with variants of these proteins.

## RESULTS

### Establishment and characterization of an ASIP-expressing B16-F10 melanoma sub-line

**Figure 1:**
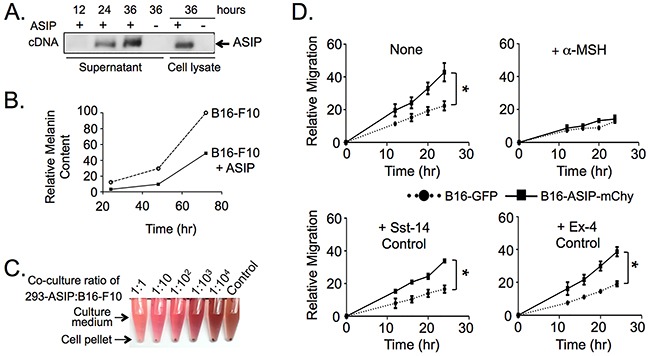
Ectopic ASIP expression inhibits MC1R in a competitive manner **A.** Western blot of culture supernatants and cell lysates from HEK 293 cells transiently expressing the mouse ASIP cDNA. Times indicate hours post-transfection. **B.** Suppression of melanin synthesis by secreted ASIP. Thirty-six hr cell-free supernatants from transfected 293 cells were added to low density B16-F10 cultures and melanin released into the media was quantified daily for three days. Values shown were normalized to the melanin content of the seventy-two hr supernatant from B16-F10 cultured in the absence of ASIP which was set to 100%. **C.** Suppression of melanin synthesis is dose dependent with respect to ASIP. Images show culture medium and cell pellets from control B16-F10 or B16-F10 co-cultured with 293 cells transiently expressing ASIP in the indicated ratios. **D.** B16-ASIP-mChy cells migrate at a faster rate than B16-GFP cells (upper left graph) in a manner that is reversed by addition of the MC1R agonist α-MSH (upper right graph, +α-MSH). Migration was not affected by addition of two control peptide hormones, somatostatin (+Sst-14) and exendin-4 (+Ex-4) whose GPCR receptors are expressed on B16-F10 cells. Shown is a representative experiment from three independent wound healing assays performed. Values are the mean ± standard deviation for triplicate cell wounds made in the same well after addition of the indicated peptide hormone. Peptide hormone concentrations were 1 μM α-MSH and Sst-14 and 10 nM Ex-4. *, p value <0.0001.

To study the effect of MC1R inhibition on melanoma engraftment and growth *in vivo* we devised a strategy that would result in local expression of ASIP as an alternative to systemic delivery of ASIP, which is predicted to have adverse effects including obesity, development of type-II diabetes, and premature infertility [30, 31]. To this end, we established an ASIP-expressing sub-line of murine B16-F10 melanoma cells, which naturally lacks endogenous ASIP expression due to transcriptional interference from homozygous insertion of a retrotransposable element in the C57BL/6 mice from which the original B16 tumor line was derived [29, 32-34]. To confirm the biological activity of mouse ASIP in our system we transfected HEK293 cells with a plasmid containing the mouse ASIP cDNA, which resulted in the expression of a 17 kDa species that reacted with anti-ASIP antiserum in both the culture supernatant and cell lysates (Figure 1A; full blot image shown in Supplementary Figure S1). When cell-free supernatants from the transfected cells were applied to B16-F10 cultures, pigment synthesis was suppressed indicating that the expressed ASIP was secreted and biologically active (Figure 1B). Likewise, in co-cultures of B16-F10 cells and ASIP cDNA-transfected HEK293 cells, but not control mock-transfected HEK293 cells, the amount of melanin pigment was reduced proportionally to the numbers of ASIP cDNA-transfected cells in the co-cultures (Figure 1C).

Next we generated stable ASIP-secreting B16-F10 cells using a lentivirus vector since calcium and lipid agent transfection efficiencies in the tumor cells were less than 15% and their intrinsic drug resistance extended to as great as 2000 μg/ml G418 and 5 μg/ml puromycin, precluding selection for ASIP expressing cells using linked drug resistance (data not shown). This oligo-clonal population of tumors cells was designated B16-ASIP, which showed low but detectable levels of ASIP secretion as judged by Western blot analysis of culture medium using anti-ASIP antiserum (data not shown). Lacking a basis for selection of this population using drug resistance markers, we identified ASIP(+) sub-clones by bioassay for inhibition of melanin synthesis and by Western blot (data not shown). One of the sub-clones showing high ASIP expression, designated B16-ASIP*, was marked with the red fluorescent protein mCherry (B16-ASIP-mChy) by a second lentivirus transduction, while parental B16-F10 cells were marked with green fluorescent protein (B16-GFP) to facilitate identification and enumeration of the two tumor cell types following implantation into mice.

We then characterized the *in vitro* migration and growth properties of the single cell sub-clonal B16-ASIP-mChy line to determine if ectopic ASIP expression affected biological properties of B16 cells known to be mediated through MC1R. We performed wound-healing assays and compared the migration rate of B16-ASIP-mChy tranduced sub-clone to the parental B16-F10 line. The ASIP-expressing cells were more mobile than parental B16-F10 (Figure 1D, upper left panel), in agreement with previous reports that addition of exogenous ASIP increases migration of B16-F1 cells [8, 27]. The increased migration we observed was evidently due to competitive inhibition of MC1R by secreted ASIP since addition of excess agonist ligand α-MSH reversed this trait (Figure 1D, upper right panel), but addition of two control agonists, the somatostatin receptor agonist somatostatin-14 (Sst-14) and the glucagon-like peptide receptor-1 agonist exendin-4 (Ex-4), which like MC1R are G protein-coupled receptors expressed by B16-F10 cells [35-37], did not (Figure 1D).

**Figure 2:**
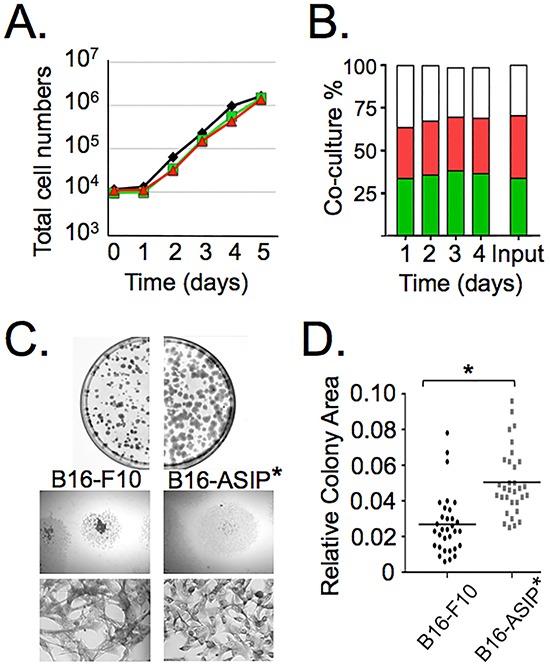
ASIP expression does not affect growth *in vitro*, but does alter colony morphology **A.** Replicate cultures of parental B16-F10 (black line), B16-GFP (green line) and B16-ASIP-mChy (red line) were seeded separately at low density and the total number of cells quantified daily for five days using a Scepter cell counter (Millipore). **B.** Replicate cultures of a 1:1:1 admixture of B16-F10:B16-GFP:B16-ASIP-mChy cells (Input) were seeded at low density and the percent of each cell type was quantified daily for four days using flow cytometry. Green bars = B16-GFP; red bars = B16-ASIP-mChy; white bars = parental B16-F10. **C.** The ASIP(-) B16-GFP cells or the B16-ASIP* cells plated at <500 cells per dish were grown for ten days then fixed and stained. The plates were photographed to show colony size and size distribution (top). Representative colonies (middle, 12.5X magnification) and representative cell morphologies within colonies (bottom, 100X magnification) are shown. **D.** Scatter plot of relative colony size (n=32). Values are the area in relative pixel units quantified from digital images using ImageJ and plotted in GraphPad Prism5. *, p value <0.0001.

We also compared the growth rate of the B16-ASIP-mChy cell line to the parental B16-F10 cells and the B16-GFP line. In contrast to previous reports that addition of exogenous ASIP had anti-proliferative effects on the B16-F1 line [8, 27], we found that the rate of B16-ASIP-mChy cell growth was comparable to the parental B16-F10 and the B16-GFP cells over a 4-5 day period in culture (Figure 2A and Supplementary Figure S2A). Even when co-cultured for 4 days in a 1:1:1 admixture of B16-F10:B16-GFP:B16-ASIP-mChy, the ratio of the three cell types remained constant (∼33% for each cell type), which is consistent with secreted ASIP having no effect on the growth rate of co-cultured ASIP-negative B16-F10 cells (Figure 2B). We confirmed that both the ASIP-mChy and the GFP-transduced lines retained MC1R expression and that MC1R was not down-regulated in the ASIP-expressing cells (data not shown).

While their proliferative rates did not differ significantly, the cultured ASIP-expressing tumor cells did show reduced heterogeneity in cell shape and particularly in colony morphology (Figure 2C). For these experiments we used the B16-ASIP* cells prior to marking with mChy. When plated at low density, the parental B16-F10 cells tended to grow on top of each other as well as grow radially outward, whereas the B16-ASIP* population grew in a more dispersed pattern and had less tendency to pile-up on top of each other. This latter trait apparently resulted in a more uniform colony size that was ∼2-fold larger in area in the ASIP-expressing population compared to parental B16-F10 colonies (Figure 2D; p-value < 0.0001, n = 32).

**Figure 3:**
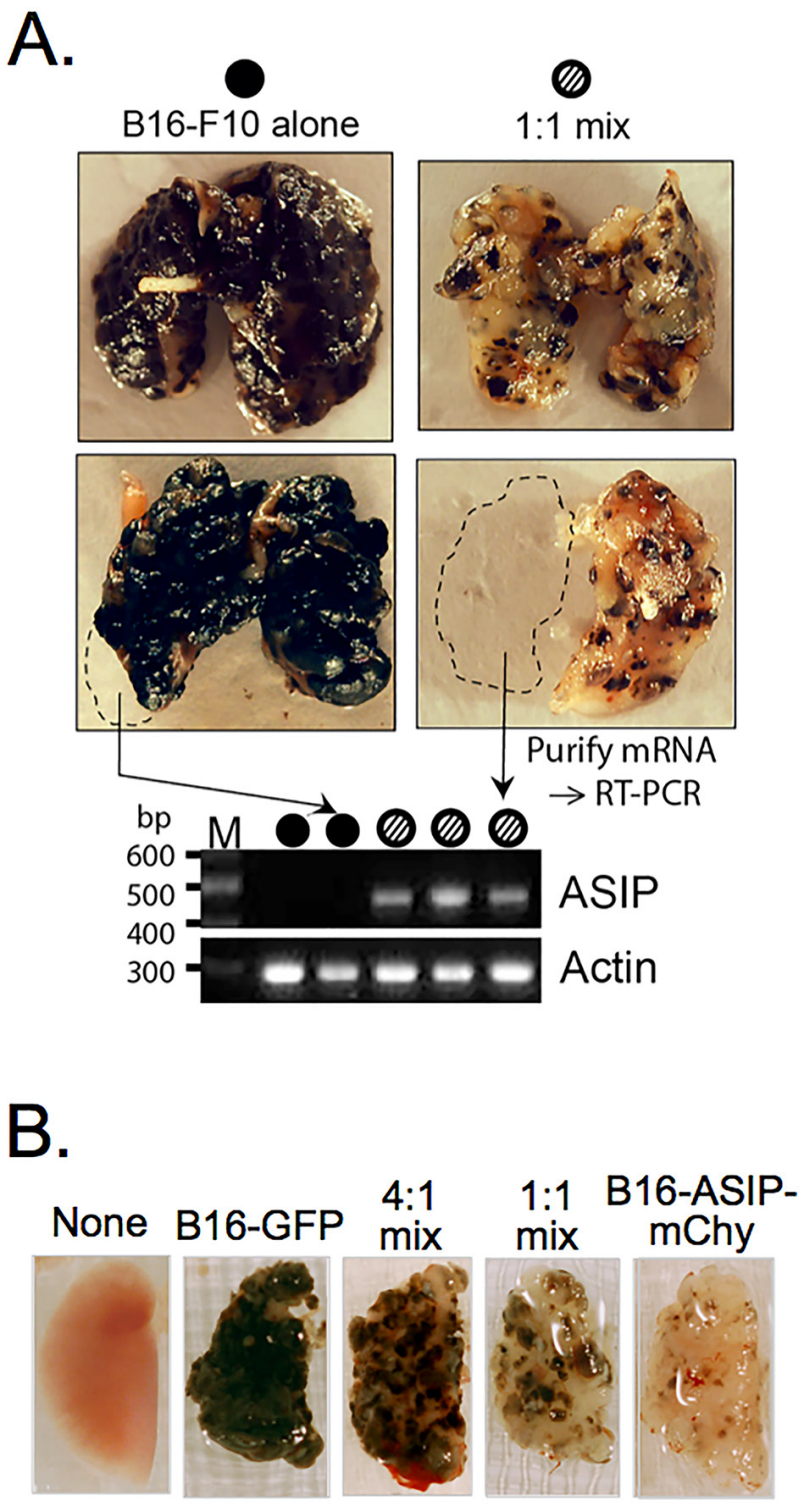
ASIP expression inhibits melanin synthesis *in vivo* **A.** Representative lungs from mice injected with 10^6^ of either parental B16-F10 cells alone (top left, n=3), a sub-population of B16-F10 selected for greater melanin production (bottom left, n=3), a 1:1 admixture of B16-F10 cells and the B16-ASIP* sub-clone that was later marked with mChy (top right, n=3), or a 1:1 admixture of B16-F10 cells and the unselected oligo-clonal population of B16-ASIP cells from which the sub-clone was derived (right bottom, n=3). Dashed lines indicate portions removed prior to fixation, which were used for mRNA isolation and RT-PCR analysis of ASIP expression. A representative agarose gel image of RT-PCR assays using mRNA of fresh lung tissue from five mice is shown below the lung images. Arrows indicate the source lung for two of the samples and filled or hashed circles indicate the mouse group. The lack of ASIP-specific products in lungs from the B16-F10 alone group confirms that parental melanoma cells and C57BL/6 lung tissue lack endogenous ASIP expression. **B.** Lung images from representative mice injected with 5 × 10^5^ B16-GFP alone, B16-ASIP-mChy alone or 4:1 and 1:1 admixtures of these two tumor cells (n=5 mice per group).

**Figure 4:**
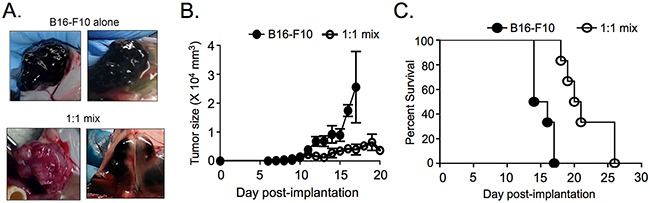
ASIP expression slows tumor growth and increases survival in a subcutaneous tumor model **A.** Representative tumor images at survival endpoint after subcutaneous injection of B16-F10 alone (n=6) or a 1:1 mix of B16-F10 and the unselected B16-ASIP population (bottom right, n=3) or its ASIP(+) sub-clone that was later marked with mChy (bottom left, n=3). **B.** Tumor size as a function of time post-implantation. **C.** Kaplan-Meier survival curves for mice bearing subcutaneous tumors (n=6 mice per group). Mice were euthanized when tumors measured >2 × 10^4^ mm^3^. Mantel-Cox analysis of mouse survival showed a 37% survival advantage for mice in the 1:1 mix (15 Day median survival for B16-F10 alone; 20.5 day median survival for 1:1 mix). p = 0.0007.

Collectively, the data shown in Figures 1 and 2 demonstrate that the ectopically expressed ASIP is secreted and is biologically active in inhibiting melanogenesis and increasing cell migration through effects on MC1R, but unlike reports that ASIP inhibited cell growth of B16-F1 cells *in vitro*, we found that the B16-ASIP-mChy cells had similar growth rates to the parental B16-F10 line. The difference between our results on growth *in vitro* with those of others may be due to the different metastatic potentials of the F1 and F10 cell lines, where the F10 line is considered more metastatic [33]. It is unlikely that the differences are due to the clonal nature of the mChy-marked cell line since the growth properties of the oligo-clonal population of B16-ASIP cells were similar to its sub-clonal B16-ASIP-mChy line (see Figures 3A and 4 below). The results from the co-culture experiment supports this idea since we observed no reduction in the growth of the parental B16-F10 cells when grown together with the ASIP-secreting cells, although we cannot discount that the difference could be due to the amount of ASIP secreted by the B16-ASIP-mChy line being insufficient to inhibit growth, yet enough to down-regulate melanogenesis and increase cell migration.

### ASIP-transduced melanoma cells inhibit melanin synthesis in a dose dependent manner *in vivo*

Although the *in vitro* properties of the sub-clonal B16-ASIP-mChy correlated with previous studies using recombinant and synthetic ASIP and were similar to its oligo-clonal B16-ASIP population, there was a possibility that when used *in vivo* the sub-clone might give ungeneralizable cell-autonomous results. To determine if this were the case, we compared the B16-ASIP* subclone, prior to marking it with mCherry, to its oligo-clonal parent B16-ASIP in a lung metastasis model and subcutaneous tumor model. These experiments would also tell us if secreted ASIP retained inverse agonist activity against MC1R-driven melanogenesis on neighboring ASIP-null tumor cells. We implanted either parental B16-F10 cells, a 1:1 admixture of B16-F10 and the oligo-clonal B16-ASIP population or a 1:1 mix of B16-F10 and the sub-clonal B16-ASIP* line by tail vein injection using the experimental lung metastasis model in syngeneic C57BL/6 mice. Mice were euthanized on various days upon displaying respiratory distress and lungs were compared for pigmented tumor formation and ASIP expression. Tumors with intense melanin pigmentation extended throughout the lungs in B16-F10 injected mice, whereas lungs from mice injected with the mixtures of the parental ASIP-null tumor cells and both the ASIP(+) oligo-clonal population and its sub-clonal derivative contained pale tumors, some with reddish-brown pheomelanin-like pigmentation, interspersed among tumors having dark eumelanin, but in greatly reduced amounts compared to parental B16-F10 tumors (Figure 3A). ASIP expression was confirmed by RT-PCR using mRNA isolated from portions of fresh lung tissue (Figure 3A, bottom panel; full gel image shown in Supplementary Figure S2B). Similar reductions in pigment were obtained from mice injected with 1:1 mixes made with either the oligo-clonal population of ASIP-transduced B16-ASIP cells (Figure 3A, bottom right image) or the sub-cloned line that was subsequently marked with mCherry (Figure 3A, top right image). Notably a similar apparent reduction in total tumor mass in lungs from mice receiving 1:1 mixtures compared to those receiving B16-F10 alone was seen regardless if the mixture contained the oligo-clonal or sub-clonal ASIP(+) tumor cells.

The suppression of pigment synthesis was dose dependent as judged by a reduction of pigment that was proportional to the number of the ASIP-positive tumor cells in the implantation mixture. The pigmentation of tumors in the lungs from mice implanted with the 4:1 and 1:1 admixtures of GFP-marked B16-F10 and mCherry-marked B16-ASIP cells (B16-ASIP-mChy) was proportional to the relative numbers of ASIP-expressing tumor cells (Figure 3B).

### Expression of ASIP increases survival in an ectopic, subcutaneous tumor model

In a second comparison of oligo-clonal and sub-clonal ASIP(+) tumors, parental B16-F10 cells alone, an equal number of a 1:1 mixture of B16-F10 and oligo-clonal B16-ASIP or sub-clonal B16-ASIP* cells were implanted subcutaneously (s.c.) into C57BL/6 mice and the effect of antagonism and inverse agonism of MC1R by ASIP on survival and tumor growth were measured. Mice were euthanized when cutaneous tumors reached 2 × 10^4^ mm^3^ in size. We found that ASIP expression correlated with reduced pigmentation within the s.c. tumors in mice receiving both 1:1 mixtures (Figure 4A) similar to that observed in the experimental lung metastasis model. Another notable difference between the parental B16-F10 tumors and those formed from the B16-ASIP cells was that while the s.c. B16-F10 tumors grew as a single tumor bed that remained intact upon excision, half the mice given the 1:1 mixes bore multiple, separated tumors that did not remain well-associated at excision. Given that ASIP did not affect the rate of growth in culture, it was unexpected to find that the 1:1 mix formed palpable mixed tumors that appeared later after implantation and were reduced in size at similar time points compared to mice administered B16-F10 cells alone (Figure 4B). The slower growth of tumors in mice implanted with the 1:1 mixtures of parental and ASIP-expressing tumor cells correlated with a significant (Mantel-Cox, p=0.0007) 37% survival advantage over mice injected with equal numbers of the parental B16-F10 cells alone (Figure 4C). Importantly increased survival and slower tumor growth were seen with mixtures of both the oligo-clonal B16-ASIP and sub-clonal B16-ASIP* cells; of the two mice that lived longest (day 26) one received the sub-clonal B16-ASIP* cells and the other the oligo-clonal cells.

Taken together these results support that the sub-clonal ASIP(+) cell line that was marked with mCherry and used in subsequent experiments is representative of the cell-autonomous behavior of an uncloned (e.g. oligo-clonal) population of B16-ASIP cells. While we favor the hypothesis that the survival advantage in the s.c. model resulted primarily from *trans* inhibition of growth of the co-injected B16-F10 cells by ASIP, the data do not rule out the possibility that the survival advantage also derived from a reduced ability of ASIP-expressing cells to establish s.c. tumors. To address this possibility, we examined tumor growth and survival in the experimental lung metastasis model since this allows for quantification of individual tumor foci as well as total tumor load.

### Inverse agonism of MC1R increases survival of mice in an experimental lung metastasis model

**Figure 5:**
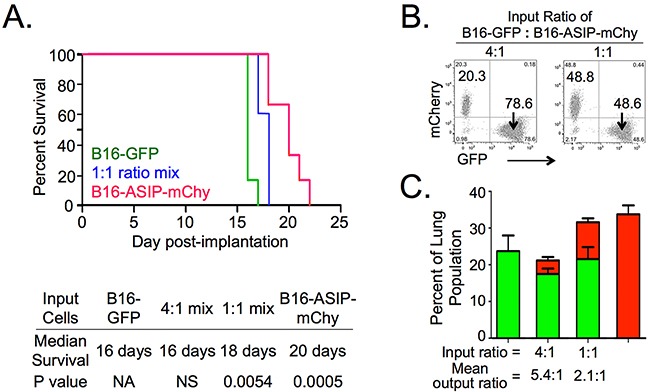
ASIP expression increases survival in an experimental lung metastasis model Groups of five mice each were injected i.v. with either B16-GFP or B16-ASIP-mChy cells individually, or in mixtures of 1:1 and 4:1 (B16-GFP:B16-ASIP-mChy), or vehicle only as controls. Mice were weighed and monitored until they exhibited respiratory distress at which point the animals were euthanized and lungs were harvested. **A.** Kaplan-Meier survival curves showing a 20% increase in median survival time in the B16-ASIP-mChy alone group and an intermediate, but still significant effect with the 1:1 group. The curve for the 4:1 mix group overlapped that of the B16-GFP group and for clarity is not shown. The median time of survival and significance (Log-rank Mantel-Cox test) for all groups is shown below the plot. **B.** Flow cytometry dot plots from tumor cell mixtures prior to i.v. injection confirm the ratios of GFP+:mChy+ cells injected on Day 0. **C.** Tumor burden at endpoint was quantified using flow cytometry of fresh lung cell suspensions. Values shown are the mean ± SEM of the percent GFP+ (green bars) or mChy+ (red bars) cells for each group (n=5 per group). For groups with mixed cell types, the mean percent mChy+ cells is shown stacked above the mean percent GFP+. Values shown for mean output ratios were calculated from the GFP+:mChy+ ratios of individual mice (Supplementary Figure S3B).

Four groups of mice (n=5) received i.v. injections of the following tumor cells: B16-GFP; B16-ASIP-mChy; and either 4:1 or 1:1 ratio mixtures of B16-GFP with B16-ASIP-mChy cells. A fifth group of mice injected with vehicle (PBS-BSA) alone served as the negative control. When each animal reached endpoint criteria for euthanasia (respiratory distress or >20% weight loss), the large left lobe of each lung was fixed and single cell suspensions were obtained from the remaining fresh lung tissue. Figure 5A shows there was a statistically significant (p-value = 0.0005), 20% increase in median survival time (16 days versus 20 days) of mice bearing the B16-ASIP-mChy tumors relative to mice with B16-GFP tumors. Mice given the 1:1 mix also had a slight, but still significant (p=0.0054) increase in survival compared to the B16-GFP cells alone, but there was no survival advantage when the ratio of B16-GFP to B16-ASIP-mChy was increased to 4:1(Figure 5A). As expected, the fixed lung lobes from mice given B16-GFP cells alone were heavily populated by melanotic tumors, whereas pigmentation was inversely proportional to the number of ASIP-secreting cells and mice injected with B16-ASIP-mChy cells alone showed little or no eumelanin pigment (Supplementary Figure S3A), suggesting that inhibition of MC1R by ASIP contributed to the longer survival observed.

To ensure that mice in each group received comparable tumor inocula of defined composition, we performed flow cytometry on the cell mixtures prior to injection (Figure 5B). Since, the 1:1 mixture was made by combining equal volumes of the B16-GFP and B16-ASIP-mChy cell suspensions, this analysis confirmed that comparable concentrations of tumor cells were present in each tumor cell suspension, suggesting that, like in the s.c. model, ASIP expression contributed to the longer survival. Interestingly, we found that when mice bearing the B16-ASIP-mChy tumors reached euthanasia criteria, they had larger tumor loads than mice bearing B16-F10 tumors (Figure 5C and Supplementary Figure S3A, p=0.0325). This trend was also seen in mice receiving the 1:1 mix. A second interesting finding was that the percentage of B16-GFP to mCherry-marked B16-ASIP cells was higher than it had been at implantation. Mice injected with a 1:1 mix reached endpoint criteria with an average 2.1:1 ratio of B16-GFP to B16-ASIP-mChy cells while those in the 4:1 mix group, which had reached endpoint two days earlier, did so with a mean ratio of 5.4:1 in their lungs (Figure 5C and Supplementary Figure S3B). These findings suggest that *in vivo*, the ASIP-expressing cells exhibited slower growth than the B16-GFP cells, which could partially explain why mice bearing B16-ASIP-mChy tumors showed increased survival, although it does not explain why the B16-ASIP-mChy and a 1:1 mix groups reached endpoint criteria with higher tumor loads.

### ASIP expression reduces tumor growth but not engraftment in the lung

To determine whether increased survival in mice receiving B16-ASIP cells resulted from reduced extravasation or engraftment capability, or was due to slower growth of the tumors in the presence of ASIP, two groups of 5 mice each were injected by tail vein with either B16-GFP alone or B16-ASIP-mChy alone. The negative control group received vehicle only. All animals were euthanized and lungs were harvested on Day 12. This time was chosen as one where tumors would be small enough that individual ones could be distinguished for enumeration using fluorescence microscopy, yet the tumor load would be sufficiently high for quantification by flow cytometry.

Since mice were injected with equal numbers of each tumor type, any differences in tumor load measured as the percentage of GFP+ or mChy+ cells in total lung cell suspensions could be due to a reduced engraftment capacity, in which case the B16-GFP group should have a greater number of individual tumors than lungs from the B16-ASIP-mChy group. Alternatively, the difference could be due to a slower growth rate of the B16-ASIP-mChy cells *in vivo*, in which case the number of individual tumors would be comparable for the B16-GFP alone and B16-ASIP-mChy alone groups.

**Figure 6:**
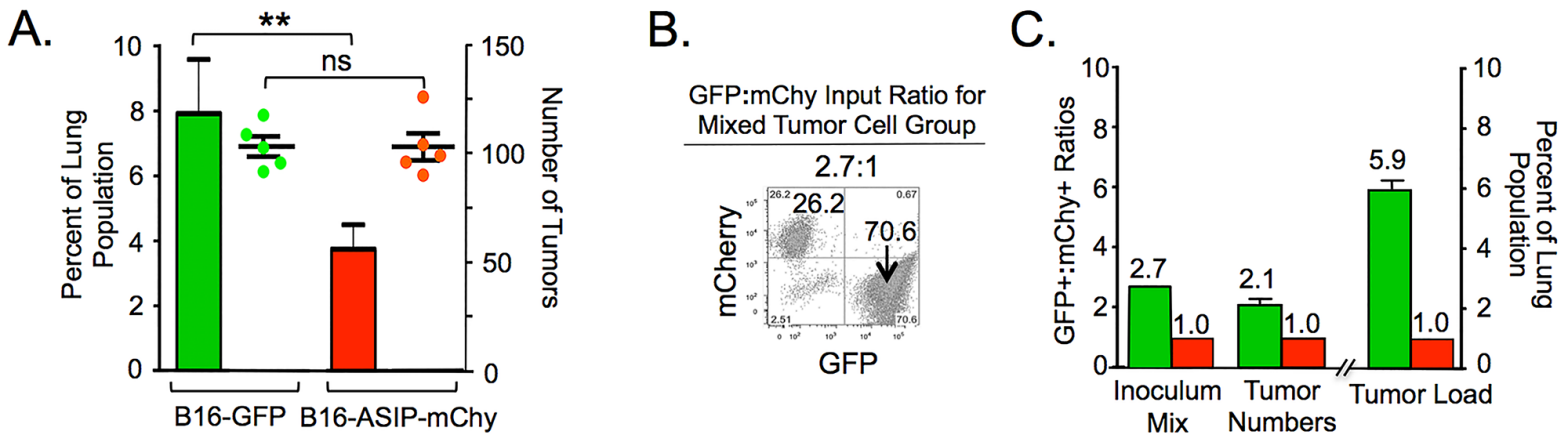
MC1R inhibition impedes the growth, but not engraftment of B16 cells in mouse lungs C57BL/6 mice (n=5/group) were injected i.v. with the indicated cell lines or with vehicle for the control group. All mice were euthanized and lungs harvested on Day 12. None of the mice exhibited respiratory distress at this time. **A.** Mean tumor load, determined by flow cytometry shown as percent B16-GFP (green bars) or B16-ASIP-mChy (red bars) in lung single cell suspensions +/− S.D. is shown. The number of tumor foci on the dorsal side of the large left lung lobe from each mouse is shown as green (B16-GFP) or red (B16-ASIP-mChy) dots +/− SEM. The number of tumors was not significantly different (ns) in the B16-GFP alone and B16-ASIP-mChy alone groups, yet tumor load in the B16-GFP group was ∼2-fold greater than in the B16-ASIP-mChy group (**p=0.0205). **B.** Flow cytometry analysis of a mixture of B16-GFP and B16-ASIP-mChy cells prior to injection into a separate set of five mice showing the mixture was at a ratio of 2.7:1, respectively. **C.** Bars indicate the ratio of B16-GFP (green) relative to B16-ASIP-mChy (red) cells in the tumor cell mixture prior to injection (Inoculum mix), the mean ratio of of GFP+ relative to mChy+ tumor foci after 12 days of growth (Tumor Numbers), and the mean percent of GFP+ or mChy+ cells in single cell suspensions from each mouse lung (Tumor Load, n=5 mice).

Tumors were counted using fluorescence micrographs of the dorsal side of each fixed left lung lobe so that the unpigmented ASIP-expressing tumors could be visualized. Tumor burden was determined by flow cytometry of fresh lung cell suspensions from the remaining lung tissue. Mice implanted with B16-GFP cells alone had an approximately 2-fold higher tumor load on Day 12 than mice bearing B16-ASIP-mChy tumors alone (Figure 6A) and this difference was statistically significant (p=0.0205). Importantly, the engraftment of ASIP-expressing cells was comparable to that of B16-GFP cells as judged by the mean number of tumors in mice implanted with each cell type alone (Figure 6A). Specifically, the similar numbers of physical tumors indicated that the 2-fold difference in tumor load was not due to reduced extravasation or engraftment efficiency of the B16-ASIP-mChy cells. We conclude instead that the tumor load difference was due to a 2-fold slower *in vivo* growth rate of the ASIP-expressing tumors.

As a test of this interpretation a group of 5 mice was injected with a mixture of tumor cell types. A negative control group received vehicle only. A roughly 3:1 mix of B16-GFP to B16-ASIP-mChy cells was chosen as one that was intermediate to the previous mixtures, which we hypothesized would be more informative of engraftment and growth capacity. The actual input ratio of the tumor inoculum was determined by flow cytometry prior to implantation to be 2.7:1 (Figure 6B). Results obtained on Day 12 from this group of mice support the interpretation that ASIP-expressing tumor cells grow significantly more slowly in mouse lungs. After 12 days of growth, the ratio of GFP+ to mChy+ tumor number was similar to the ratio of cells used for implantation, consistent with comparable engraftment capacity (Figure 6C and Supplementary Figure S4B). However, in determining tumor load by flow cytometry there was ∼6-fold more GFP+ than mChy+ cells (Figure 6C and Supplementary Figure S4C), which corresponded to an ∼2-fold increase in the GFP+:mChy+ tumor cell load relative to the input ratio. Taken together these findings indicate that the *in vivo* doubling time for ASIP-expressing melanoma cells was approximately 2-fold longer than that of ASIP-negative tumor cells.

**Figure 7:**
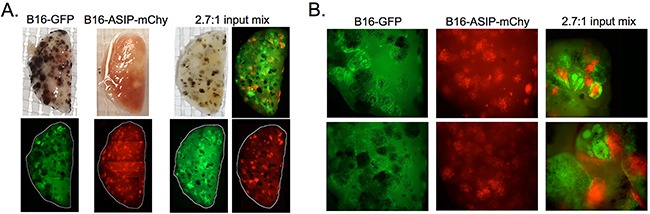
**A.** Photographs and composite images of fluorescence micrographs of representative fixed lung lobes from the indicated groups of mice whose outcome data are shown in Figure 6. White lines in the fluorescence images indicate the perimeter of lung lobes which were assembled from individual 12.5X magnification images as delineated in Supplementary Figure S4A. The composite image in the upper right of the 2.7:1 input mix is a merged fluorescence image from the two images shown on the bottom. **B.** Enlarged images from two representative mice each in the B16-GFP, B16-ASIP-mChy (12.5X magnification) and 2.7:1 mix groups (top at 25X and bottom at 50X magnification) are shown.

Consistent with their slower rate of growth, B16-ASIP-mChy tumors appeared smaller in size (Figure 7). The B16-GFP tumor masses were distinctly larger and contained more dense and heterogeneous arrangements of tumor cells, while the B16-ASIP-mChy cells formed tumors that appeared smaller and had a more uniform cell distribution within the tumors. The tumor morphology was highly reminiscent of the more homogeneous colony shape seen when the B16-ASIP cells were grown *in vitro*.

## DISCUSSION

In this report we describe the generation of B16 cells that stably express the MC1R inverse agonist ASIP in amounts sufficient to suppress melanogenesis when these cells are grown in culture, as well as when introduced into animals either intravenously or when implanted subcutaneously. The most unanticipated result from our studies was that chronic expression of ASIP within the tumor microenvironment provided a survival advantage to mice bearing both subcutaneous tumors and experimental lung metastases. This survival advantage was due, as least in part, to a slower rate of growth of the ASIP-expressing cells, which was not observed when the cells were grown in culture. There was no difference in engraftment efficiency as determined by enumeration of physical tumors found in the lungs after systemic administration, but a notable difference was that the individual ASIP-secreting tumors were smaller and more dispersed throughout the lung and dermal tissues compared to either the parental B16-F10 tumors or those formed by the B16-GFP cells, which were generally larger, more densely packed, and more heterogeneously dispersed within tumors.

While MC1R is well-known as a normal human melanocyte growth receptor (reviewed in [38]), it has not previously been shown to be an important growth receptor for melanoma. Earlier studies examining the effect of MC1R signaling on melanoma cell culture proliferation were inconsistent, with some reports observing no effect while others describing modest effects on growth [4, 8, 10, 27]. Given the effects on tumor growth *in vivo*, but lack of an effect *in vitro* reported here, the confounding results from the earlier studies may simply have been due to variability in culture conditions or the sub-lines of B16 cells used. A role for MC1R as a growth receptor for melanoma is in agreement with transcriptome analysis of a human melanoma cell line in which MC1R was inhibited by transient transfection of an ASIP cDNA. In that report the translation initiation factor eIF-4B was identified among several genes transcriptionally down-regulated in the ASIP-expressing tumor cell cultures [10]. eIF-4B is a subunit of the cap-dependent translation initiation complex which is regulated by mTOR to increase translation rates of mRNAs with secondary structure in their 5′ untranslated region, and which is required for proliferation of normal and malignant cells (reviewed in [39]). Depletion of eIF-4B using silencing RNA in the human cervical cancer line HeLa reduced proliferation by greater than 90% over a five day period and rendered them more sensitive to killing by the topoisomerase inhibitor camptothecin [40]. It is thereby reasonable to speculate that MC1R signaling participates in maintaining levels of eIF-4B protein high enough to support robust melanoma cell growth and division.

While we did not observe a difference in engraftment capacity for ASIP-expressing B16-F10 cells, Seong and colleagues observed a decrease in melanin-positive B16-F10 lung tumors when an anti-MC1R silencing RNA was introduced just prior to i.v. injection [41]. These authors concluded that MC1R is required for efficient extravasation into the lung. However, this conclusion was based on enumeration of pigmented tumors and did not take into account that loss of MC1R expression would inhibit melanogenesis [20, 42] resulting in unpigmented tumors which would not be included in the enumeration. We suspect that if the tumor cells had been marked with a fluorescent protein and tumors enumerated in fluorescent micrographs that no difference in tumor number would have been seen.

Another possible mechanism affecting growth inhibition in ASIP-expressing cells may be contributed by the tumor suppressor PTEN. In normal melanocytes α-MSH induces PTEN to associate with the cytoplasmic domain of wild type MC1R, an association that protects PTEN from ubiquitinylation and degradation [43]. Interestingly MC1R variants with reduced affinity for α-MSH show a reduced association with, and protection of, PTEN [43], suggesting that ASIP may inhibit MC1R protection of PTEN and in this manner slow tumor growth.

The second unexpected finding from our studies was that although the ASIP-expressing tumors were generally smaller in size, mice implanted with either B16-ASIP-mChy cells alone, or with a 1:1 mixture of B16-GFP and B16-ASIP-mChy cells had significantly larger overall tumor burdens at the time of euthanasia than did mice with either a 4:1 mix or B16-GFP cells only. While the smaller size of the B16-ASIP-mChy tumors likely results from the slower growth of these cells, as indicated by the increase in the ratio of GFP(+) cells to mChy(+) cells recovered from mice relative to the input ratios, this growth differential alone could not account for the increased overall tumor burdens at survival endpoints. If growth rate were the sole factor determining survival we would have expected the tumor burden to be the same when mice began to exhibit respiratory distress and were euthanized, but this was not the case.

Why then did the mice receiving either i.v. B16-ASIP-mChy alone, or a 1:1 mixture of B16-GFP and B16-ASIP mChy cells live longer before reaching euthanasia criteria? One relevant observation was that when lungs were harvested on Day 12 the tumor foci in the parental B16-implanted lungs were larger and more heterogeneously dispersed than the B16-ASIP tumor foci (Figure 7B). We favor the explanation that the B16-F10 tumors compressed the alveoli to a greater extent per tumor dose with the result that animals exhibited respiratory distress and reached euthanasia criteria sooner. The greater tumor load found in the B16-ASIP-mChy bearing lungs (Figure 5C) may have been better tolerated because the smaller ASIP-expressing tumors exerted less restriction on pulmonary function.

A second possibility is that ASIP provides a protective function that helps maintain lung function in the presence of a higher tumor load. Conversely, α-MSH made by the parental B16 and the B16-GFP cells may negatively affect lung function. For example, ASIP may influence survival and *in vivo* tumor growth by inhibition of MC1R found on the tumor vasculature. Although not studied in melanoma, α-MSH signaling through MC1R on vascular endothelial cells increases expression and phosphorylation of endothelial NO synthase and the subsequent production of nitric oxide (NO) in the mouse aorta and coronary vasculature [44]. If the tumor vasculature is similarly responsive to α-MSH agonism then ASIP secreted from tumor cells may counter this influence and decrease tumor blood supply.

A third possible mechanism might involve the differential distribution of ASIP-secreting tumors compared to ASIP-negative parental B16-F10 tumors. This likely results from the increased migratory behavior that we observed in B16 cells stably expressing ASIP as measured in the wound-healing assay (Figure 1). This effect has been reported by others when recombinant ASIP was added to B16 cells in culture [27], or when human ASIP was transiently expressed in human melanoma cells [10], and it was proposed that the increased wound healing upon acute ASIP exposure would correlate with a higher metastatic potential [27], but this has not been tested. While we found that mice given mixtures of B16-GFP and B16-ASIP-mChy cells intravenously had “metastases” at various locations other than the pleural cavity, with secondary sites being predominately found in the kidneys, ovaries, and lymph nodes (our unpublished observations and [33]), the B16-ASIP-mChy cells did not show a higher propensity to form secondary site metastases than the B16-GFP cells. Therefore, the increased migratory behavior of ASIP-expressing cells appeared to be limited to local spread within the lung or subcutaneous tissue and did not represent a true increase in metastatic potential. However, a more thorough analysis using larger numbers of animals is needed to confirm these observations.

As a potential new target for melanoma therapy, mimetic peptides derived from ASIP may be useful in biased inhibition of pro-melanoma functions of MC1R although only a few variants of ASIP have been examined. Another potential therapeutic is a peptide derived from human β-defensin 3 (HBD3) which is a natural but neutral antagonist of MC1R and MC4R that competes with and antagonizes α-MSH and ASIP to neutralize cAMP signaling through these receptors [45]. Analysis of a large set of peptide variations of HBD3 yielded one called Neut 2 whose Ki for MC1R (422 nM) was roughly four-fold lower than its Ki for MC4R (1568 nM) [46]. This difference presents a potential therapeutic window for neutral antagonism of MC1R in the absence of an appreciable effect on MC4R control of appetite in the brain. Another highly desirable trait of a therapeutic MC1R ligand would be to inhibit its growth receptor activity on melanoma cells without interfering with anti-inflammatory signaling through MC1R on macrophages. One such ligand is the orally administered small molecule ligand AP1189, which has been shown to compete for the α-MSH binding site on MC1R to reduce cytokine release by macrophages in an experimental inflammatory arthritis mouse model, but did not evoke cAMP or melanogenesis in B16-F10 melanoma cultures [47]. While not yet tested, it may also inhibit growth receptor signaling through MC1R.

All of the analyses described in this report relied on our ability to quantify tumor loads in the lung in the absence of melanin synthesis. Tumor load with the B16 model is typically quantified by counting individual tumor foci made visible by high levels of melanin synthesis. However, the lack of pigmentation in the ASIP(+) cells prevented us from accurately visualizing tumors arising from the B16-ASIP cells. To overcome this limitation we generated B16 variants that expressed either GFP for the parental cell type or mCherry for the B16-ASIP cells. By marking these cells with the fluorescent reporter we could identify both micro- and macro-tumor foci using fluorescence microscopy as has been done by others [48-51], but the most significant advantage of using the two fluorescent reporters to individually mark the two cell types was the ability to use flow cytometry to quantify tumor load. The application of flow cytometry also allowed us to detect the 2-fold difference in the growth rate of ASIP(+) and ASIP-null cells after recovery from the lungs of animals, which otherwise would have been difficult, if not impossible to ascertain. Importantly for the studies reported here, we could also examine the influence of secreted ASIP on the distribution and morphology of adjacent ASIP-null tumors. Many other groups have generated B16 lines that express GFP and/or RFP, but to our knowledge we are the first to have successfully taken advantage of these reporters to examine changes in the physiology of cells through the use of flow cytometry and fluorescence microscopy. By visualizing tumor morphology and interactions with the surrounding stroma, it should be possible to study the response to various anti-tumor agents and the contributions of host factors that either inhibit or promote metastasis.

## MATERIALS AND METHODS

### Cell lines, ASIP cDNA and lentiviral vectors

Human HEK 293 (ATCC), mouse B16-F10 melanoma (ATCC) and the derivative cell lines described below were cultured in high glucose Dulbecco's modified Eagle medium (DMEM) containing 8% FBS. A mouse ASIP cDNA (gift of Dr. R. Mynatt, Penington Biomedical Research Center) was subcloned into mammalian expression vector pCAGGS [52] prior to transfection into HEK293 cells using Lipofectamine 2000 according to the manufacturer's instructions. The ASIP, GFP or mCherry cDNAs were inserted into an FUW lentiviral transfer genome (Addgene) in which the ubiquitin C promoter was replaced by the eukaryotic elongation factor 1-alpha promoter and packaged into VSV-G pseudotyped lentiviral vectors using a ViraPower kit (Invitrogen) according to the manufacturer's instructions. B16-F10 cells were transduced with the ASIP-lentivirus vector to generate a stable, oligo-clonal population of ASIP-secreting cells designated B16-ASIP. After limiting dilution cloning, multiple subclonal lines were identified that expressed detectable amounts of ASIP in the culture medium and one, designated B16-ASIP*, was selected at random for use in the studies. To facilitate identification and enumeration of tumor cells, the parental B16-F10 cells were marked with green fluorescent protein using lentiviral transduction and the stable ASIP-secreting subclone was marked with the red fluorescent protein mCherry (mChy) by a second lentivirus transduction, then both populations were sorted for high fluorescent protein (GFP or mChy) expression by FACS and designated B16-GFP and B16-ASIP-mChy, respectively.

### Western blot analysis and quantification of melanin

For Western blot analysis of secreted ASIP, detached cells and debris were removed from culture medium by centrifugation and 50 μL of the cleared supernatant was electrophoresed on a 9% SDS-polyacrylamide gel under reducing conditions and then analyzed by immunoblot probed with anti-mouse ASIP antiserum (generous gift from Dr. V. J. Hearing, NIH) using ECL chemiluminescence (Pierce). Cell lysates were prepared by washing confluent monolayers with PBS to remove excess serum albumin and lysing in MNT buffer [40 mM 2-(N-morpholino)ethanesulfonic acid (MES), 60 mM Tris, 200 mM NaCl, 2.5 mM EDTA, adjusted to pH 7.4] containing 1% Triton X-100. Proteins from approximately 7×10^4^ cell-equivalents were separated by SDS-PAGE and subjected to immunoblot analysis as described above.

The relative melanin content in cell culture supernatants was measured as described by Watts and colleagues [53] except that absorbance at 355 nm was used because spectra measured on serial dilutions of purified melanin pigment (Sigma-Aldrich, catalog #M8631) prepared in growth medium showed an absorbance peak at 355 nm which was directly proportional to the melanin concentration.

### Determination of in vitro colony morphology and cell migration assay

B16-F10 or B16-ASIP were plated at a density of 500 - 1000 cells in 60 mm culture dishes, fed on day 3, incubated for 7 more days, then fixed and stained on day 10 with Modified Wright-Giemsa (Sigma-Aldrich, catalog #WG-16). Color images of each plate were captured using a D50 digital camera (Nikon) mounted on a SZ-PT microscope (Olympus) and the relative area of thirty-two random colonies (i.e. the first thirty-two individual colonies encountered in a horizontal scan starting at the approximate equator of each plate) were measured using ImageJ. The mean area ± standard deviation and significance of differences in relative colony size were determined using unpaired t-tests in GraphPad Prism5. For the wound-healing migration assays, B16-GFP or B16-ASIP-mChy cells were plated at 2 × 10^5^ cells per well of a 24-well plate and incubated for 8 hr to promote attachment, the monolayers were scratched without removing the medium using a sterile Avant® 20 μL pipette tip. Images were captured immediately after introduction of the scratch/wound and at 12, 16, 20 and 24 hr post-wounding using a Zeiss AxioVert fluorescence microscope fitted with a color Zeiss AxioCam camera and Axiovision 4.7 software. Peptide hormones or control PBS were added to the medium immediately after wounding at the following final concentrations: 1 μM α-MSH, 1 μM somatostatin-14, or 10 nM exendin-4. The extent of cell migration was quantified by measuring the open area in the images at each time point using ImageJ analysis software and the values were plotted and analyzed using GraphPad Prism5.

### RT-PCR

Total RNA was isolated from cultured cells, dissociated lung and subcutaneous tumor tissues using QiaAmp RNA Blood kit (Qiagen). cDNA templates were synthesized using Transcriptor First Strand cDNA Synthesis Kit (Roche) followed by polymerase chain amplification using GoTaq Flexi DNA Polymerase (Promega). Sequences of primers used for amplification will be provided upon request.

### Mice and tumor implantation

Animal welfare and procedures were performed strictly in accordance with UTHSC Institutional Animal Use and Care committee approved protocols. Female C57BL/6 mice (7-10 weeks old, Charles River) were used in all studies. For injection into study mice, tumor cells were grown to 80% confluence, detached by trypsin, washed once with growth medium, once with 0.5% BSA in PBS lacking magnesium and calcium (PBS-BSA), and then suspended in ice-cold PBS-BSA to prevent cell clumping and maintain cell viability prior to injection. Subcutaneous (s.c.) injection of 5×10^5^ total tumor cells into the right hind flank or tail vein injection (i.v.) of 10^6^ or 5×10^5^ total cells were used to establish tumor models. Once s.c. tumors became palpable, their length and width in millimeters were measured using micro-calipers and tumor volume was calculated using the modified ellipsoidal formula [*tumor volume* = 0.52(*length* × *width*^2^)]. Mice were euthanized when tumor volumes exceeded 2 × 10^4^ mm^3^, grooming became poor, >20% of starting weight was lost, or if the tumors became ulcerated. In some lung metastasis studies, mice were euthanized at defined times post-implantation. In survival studies, i.v.-injected mice were euthanized when they showed signs of respiratory distress, poor grooming or >20% weight loss.

### Tumor burden determination in experimental lung metastases

After euthanasia by deep sedation with ketamine/xylazine (200 mg/kg ketamine and 20 mg/kg xylazine) followed by exsanguination and perfusion with ice-cold saline (0.9% NaCl in calcium and magnesium-free PBS), the heart and lungs were removed *in toto* and the left lung was excised and placed in 4% paraformaldehyde. The heart was discarded and the remaining lung tissue was placed in ice-cold DMEM with 5% FBS and single cell suspensions were prepared using a MACS Tumor Dissociation Kit-mouse (Miltenyi Biotec) according to the manufacturer's instructions with the following modifications to increase tumor cell dissociation and reduce their retention in fibrous material on the cell strainers. Lungs were minced much finer than the manufacturer's recommendation, with particular attention paid to cutting apart individual tumor nodules, the dissociation cocktail was added at room temperature, the tumor dissociation program was run twice and the incubation at 37°C was carried out for 1.5 hr. Tumor burden was then quantified using two methods. First, cell suspensions obtained by dissociation of lung tissue were analyzed by flow cytometry. Second, tumor numbers were quantified from fluorescence micrographs using image analysis software. Composite fluorescence images (Supplementary Figure S4A) were assembled from overlapping green and red fluorescent micrographs captured in 200 msec exposures at 1.25x magnification (2.5x objective and 0.5x reductive camera coupler) using a Zeiss AxioVert epifluorescence microscope and color Zeiss AxioCam digital camera with Axiovision 4.7 software. While densely pigmented B16-GFP tumors showed little green fluorescent signal, in agreement with incident excitation light absorption by the eumelanin as previously reported for GFP-marked B16-F10 tumors *in vivo* [51], their identity as pigmented tumors were confirmed by comparison with matching color micrographs (Figure 7A). Images of B16-GFP alone and B16-ASIP-mChy alone mouse lungs were scored for tumor number and morphology without adjustment; images shown are also unadjusted. Those of the 2.7:1 mix group were adjusted uniformly in batches to the level of fluorescence intensity observed through the binocular eyepieces on the microscope using an automated action script in PhotoShop CC (Adobe) prior to quantification of tumor numbers. Tumor numbers obtained from this quantification were graphed using GraphPad Prism5.

### Statistical analyses

Statistical analyses were performed using the GraphPad Prism5 software and the following statistical algorithms to calculate P values and significance: Mantel-Cox Log-rank Test (survival curves); unpaired, 2-tailed t-test (mean number GFP^+^ and mChy^+^ tumors in lungs); unpaired t-test (mean colony size) and 2-tailed t-test with 99% confidence interval throughout (migration assays).

## SUPPLEMENTARY FIGURES



## References

[1] Bonilla C, Boxill LA, Donald SA, Williams T, Sylvester N, Parra EJ, Dios S, Norton HL, Shriver MD, Kittles RA (2005). The 8818G allele of the agouti signaling protein (ASIP) gene is ancestral and is associated with darker skin color in African Americans. Hum Genet.

[2] Han J, Kraft P, Nan H, Guo Q, Chen C, Qureshi A, Hankinson SE, Hu FB, Duffy DL, Zhao ZZ, Martin NG, Montgomery GW, Hayward NK, Thomas G, Hoover RN, Chanock S (2008). A genome-wide association study identifies novel alleles associated with hair color and skin pigmentation. PLoS Genet.

[3] Miller MW, Duhl DM, Vrieling H, Cordes SP, Ollmann MM, Winkes BM, Barsh GS (1993). Cloning of the mouse agouti gene predicts a secreted protein ubiquitously expressed in mice carrying the lethal yellow mutation. Genes Dev.

[4] Hida T, Wakamatsu K, Sviderskaya EV, Donkin AJ, Montoliu L, Lynn Lamoreux M, Yu B, Millhauser GL, Ito S, Barsh GS, Jimbow K, Bennett DC (2009). Agouti protein, mahogunin, and attractin in pheomelanogenesis and melanoblast-like alteration of melanocytes: a cAMP-independent pathway. Pigment Cell Melanoma Res.

[5] Hunt G, Thody AJ (1995). Agouti protein can act independently of melanocyte-stimulating hormone to inhibit melanogenesis. J Endocrinol.

[6] Ollmann MM, Lamoreux ML, Wilson BD, Barsh GS (1998). Interaction of Agouti protein with the melanocortin 1 receptor in vitro and in vivo. Genes Dev.

[7] Patel MP, Cribb Fabersunne CS, Yang YK, Kaelin CB, Barsh GS, Millhauser GL (2010). Loop-swapped chimeras of the agouti-related protein and the agouti signaling protein identify contacts required for melanocortin 1 receptor selectivity and antagonism. J Mol Biol.

[8] Siegrist W, Drozdz R, Cotti R, Willard DH, Wilkison WO, Eberle AN (1997). Interactions of alpha-melanotropin and agouti on B16 melanoma cells: evidence for inverse agonism of agouti. J Recept Signal Transduct Res.

[9] Levy C, Khaled M, Fisher DE (2006). MITF: master regulator of melanocyte development and melanoma oncogene. Trends Mol Med.

[10] Voisey J, Kelly G, Van Daal A (2003). Agouti signal protein regulation in human melanoma cells. Pigment Cell Res.

[11] Taylor NJ, Reiner AS, Begg CB, Cust AE, Busam KJ, Anton-Culver H, Dwyer T, From L, Gallagher RP, Gruber SB, Rosso S, White KA, Zanetti R, Orlow I, Thomas NE, Rebbeck TR (2015). Inherited variation at MC1R and ASIP and association with melanoma-specific survival. Int J Cancer.

[12] Williams PF, Olsen CM, Hayward NK, Whiteman DC (2011). Melanocortin 1 receptor and risk of cutaneous melanoma: a meta-analysis and estimates of population burden. Int J Cancer.

[13] Chatzinasiou F, Lill CM, Kypreou K, Stefanaki I, Nicolaou V, Spyrou G, Evangelou E, Roehr JT, Kodela E, Katsambas A, Tsao H, Ioannidis JP, Bertram L, Stratigos AJ (2011). Comprehensive field synopsis and systematic meta-analyses of genetic association studies in cutaneous melanoma. J Natl Cancer Inst.

[14] Helsing P, Nymoen DA, Rootwelt H, Vardal M, Akslen LA, Molven A, Andresen PA (2012). MC1R, ASIP, TYR, and TYRP1 gene variants in a population-based series of multiple primary melanomas. Genes Chromosomes Cancer.

[15] Goldstein AM, Landi MT, Tsang S, Fraser MC, Munroe DJ, Tucker MA (2005). Association of MC1R variants and risk of melanoma in melanoma-prone families with CDKN2A mutations. Cancer Epidemiol Biomarkers Prev.

[16] Hill VK, Gartner JJ, Samuels Y, Goldstein AM (2013). The genetics of melanoma: recent advances. Annu Rev Genomics Hum Genet.

[17] Marzuka-Alcala A, Gabree MJ, Tsao H (2014). Melanoma susceptibility genes and risk assessment. Methods Mol Biol.

[18] Duffy DL, Zhao ZZ, Sturm RA, Hayward NK, Martin NG, Montgomery GW (2010). Multiple pigmentation gene polymorphisms account for a substantial proportion of risk of cutaneous malignant melanoma. J Invest Dermatol.

[19] Maccioni L, Rachakonda PS, Scherer D, Bermejo JL, Planelles D, Requena C, Hemminki K, Nagore E, Kumar R (2013). Variants at chromosome 20 (ASIP locus) and melanoma risk. Int J Cancer.

[20] Valverde P, Healy E, Jackson I, Rees JL, Thody AJ (1995). Variants of the melanocyte-stimulating hormone receptor gene are associated with red hair and fair skin in humans. Nat Genet.

[21] Raimondi S, Sera F, Gandini S, Iodice S, Caini S, Maisonneuve P, Fargnoli MC (2008). MC1R variants, melanoma and red hair color phenotype: a meta-analysis. Int J Cancer.

[22] Chung H, Lee JH, Jeong D, Han IO, Oh ES (2012). Melanocortin 1 receptor regulates melanoma cell migration by controlling syndecan-2 expression. J Biol Chem.

[23] Scott G, Cassidy L, Abdel-Malek Z (1997). Alpha-melanocyte-stimulating hormone and endothelin-1 have opposing effects on melanocyte adhesion, migration, and pp125FAK phosphorylation. Exp Cell Res.

[24] Sviderskaya EV, Hill SP, Balachandar D, Barsh GS, Bennett DC (2001). Agouti signaling protein and other factors modulating differentiation and proliferation of immortal melanoblasts. Dev Dyn.

[25] Cheli Y, Giuliano S, Botton T, Rocchi S, Hofman V, Hofman P, Bahadoran P, Bertolotto C, Ballotti R (2011). Mitf is the key molecular switch between mouse or human melanoma initiating cells and their differentiated progeny. Oncogene.

[26] Murata J, Ayukawa K, Ogasawara M, Fujii H, Saiki I (1997). Alpha-melanocyte-stimulating hormone blocks invasion of reconstituted basement membrane (Matrigel) by murine B16 melanoma cells. Invasion Metastasis.

[27] Le Pape E, Passeron T, Giubellino A, Valencia JC, Wolber R, Hearing VJ (2009). Microarray analysis sheds light on the dedifferentiating role of agouti signal protein in murine melanocytes via the Mc1r. Proc Natl Acad Sci U S A.

[28] Siegrist W, Willard DH, Wilkison WO, Eberle AN (1996). Agouti protein inhibits growth of B16 melanoma cells in vitro by acting through melanocortin receptors. Biochem Biophys Res Commun.

[29] Bultman SJ, Klebig ML, Michaud EJ, Sweet HO, Davisson MT, Woychik RP (1994). Molecular analysis of reverse mutations from nonagouti (a) to black-and-tan (a(t)) and white-bellied agouti (Aw) reveals alternative forms of agouti transcripts. Genes Dev.

[30] Klebig ML, Wilkinson JE, Geisler JG, Woychik RP (1995). Ectopic expression of the agouti gene in transgenic mice causes obesity, features of type II diabetes, and yellow fur. Proc Natl Acad Sci U S A.

[31] Yen TT, Gill AM, Frigeri LG, Barsh GS, Wolff GL (1994). Obesity, diabetes, and neoplasia in yellow A(vy)/− mice: ectopic expression of the agouti gene. Faseb j.

[32] Fidler IJ, Kripke ML (1977). Metastasis results from preexisting variant cells within a malignant tumor. Science.

[33] Fidler IJ, Nicolson GL (1976). Organ selectivity for implantation survival and growth of B16 melanoma variant tumor lines. J Natl Cancer Inst.

[34] Fidler IJ, Nicolson GL (1978). Tumor cell and host properties affecting the implantation and survival of blood-borne metastatic variants of B16 melanoma. Isr J Med Sci.

[35] Akihisa T, Watanabe K, Yamamoto A, Zhang J, Matsumoto M, Fukatsu M (2012). Melanogenesis inhibitory activity of monoterpene glycosides from Gardeniae Fructus. Chem Biodivers.

[36] Goke R, Fehmann HC, Linn T, Schmidt H, Krause M, Eng J, Goke B (1993). Exendin-4 is a high potency agonist and truncated exendin-(9-39)-amide an antagonist at the glucagon-like peptide 1-(7-36)-amide receptor of insulin-secreting beta-cells. J Biol Chem.

[37] Szende B, Keri G (2003). TT-232: a somatostatin structural derivative as a potent antitumor drug candidate. Anticancer Drugs.

[38] Abdel-Malek Z, Scott MC, Suzuki I, Tada A, Im S, Lamoreux L, Ito S, Barsh G, Hearing VJ (2000). The melanocortin-1 receptor is a key regulator of human cutaneous pigmentation. Pigment Cell Res.

[39] Shahbazian D, Parsyan A, Petroulakis E, Hershey J, Sonenberg N (2010). eIF4B controls survival and proliferation and is regulated by proto-oncogenic signaling pathways. Cell Cycle.

[40] Shahbazian D, Parsyan A, Petroulakis E, Topisirovic I, Martineau Y, Gibbs BF, Svitkin Y, Sonenberg N (2010). Control of cell survival and proliferation by mammalian eukaryotic initiation factor 4B. Mol Cell Biol.

[41] Seong I, Min HJ, Lee JH, Yeo CY, Kang DM, Oh ES, Hwang ES, Kim J (2012). Sox10 controls migration of B16F10 melanoma cells through multiple regulatory target genes. PLoS One.

[42] Robbins LS, Nadeau JH, Johnson KR, Kelly MA, Roselli-Rehfuss L, Baack E, Mountjoy KG, Cone RD (1993). Pigmentation phenotypes of variant extension locus alleles result from point mutations that alter MSH receptor function. Cell.

[43] Cao J, Wan L, Hacker E, Dai X, Lenna S, Jimenez-Cervantes C, Wang Y, Leslie NR, Xu GX, Widlund HR, Ryu B, Alani RM, Dutton-Regester K, Goding CR, Hayward NK, Wei W (2013). MC1R is a potent regulator of PTEN after UV exposure in melanocytes. Mol Cell.

[44] Rinne P, Nordlund W, Heinonen I, Penttinen AM, Saraste A, Ruohonen ST, Makela S, Vahatalo L, Kaipio K, Cai M, Hruby VJ, Ruohonen S, Savontaus E (2013). alpha-Melanocyte-stimulating hormone regulates vascular NO availability and protects against endothelial dysfunction. Cardiovasc Res.

[45] Beaumont KA, Smit DJ, Liu YY, Chai E, Patel MP, Millhauser GL, Smith JJ, Alewood PF, Sturm RA (2012). Melanocortin-1 receptor-mediated signalling pathways activated by NDP-MSH and HBD3 ligands. Pigment Cell Melanoma Res.

[46] Nix MA, Kaelin CB, Ta T, Weis A, Morton GJ, Barsh GS, Millhauser GL (2013). Molecular and functional analysis of human beta-defensin 3 action at melanocortin receptors. Chem Biol.

[47] Montero-Melendez T, Gobbetti T, Cooray SN, Jonassen TE, Perretti M (2015). Biased agonism as a novel strategy to harness the proresolving properties of melanocortin receptors without eliciting melanogenic effects. J Immunol.

[48] Bobek V, Kolostova K, Pinterova D, Boubelik M, Gurlich R, Hoffman RM (2011). Tail spontaneous metastatic mouse model: comparison of metastatic potential of orthotopic and heterotopic models imaged by GFP and RFP protein. In Vivo.

[49] Moore A, Sergeyev N, Bredow S, Weissleder R (1998). A model system to quantitate tumor burden in locoregional lymph nodes during cancer spread. Invasion Metastasis.

[50] Wunderbaldinger P, Josephson L, Bremer C, Moore A, Weissleder R (2002). Detection of lymph node metastases by contrast-enhanced MRI in an experimental model. Magn Reson Med.

[51] Yang M, Jiang P, An Z, Baranov E, Li L, Hasegawa S, Al-Tuwaijri M, Chishima T, Shimada H, Moossa AR, Hoffman RM (1999). Genetically fluorescent melanoma bone and organ metastasis models. Clin Cancer Res.

[52] Niwa H, Yamamura K, Miyazaki J (1991). Efficient selection for high-expression transfectants with a novel eukaryotic vector. Gene.

[53] Watts KP, Fairchild RG, Slatkin DN, Greenberg D, Packer S, Atkins HL, Hannon SJ (1981). Melanin content of hamster tissues, human tissues, and various melanomas. Cancer Res.

