# Supplementary material for: Inhibition of melanocortin 1 receptor slows melanoma growth, reduces tumor heterogeneity and increases survival
Source: Oncotarget. 2016 Mar 25;7(18):26331–45. doi: 10.18632/oncotarget.8372 (PMC5041983; doi:10.18632/oncotarget.8372)
Supplement: Supplementary file 1 [file oncotarget-07-26331-s001.pdf]

SUPPLEMENTARY FIGURES

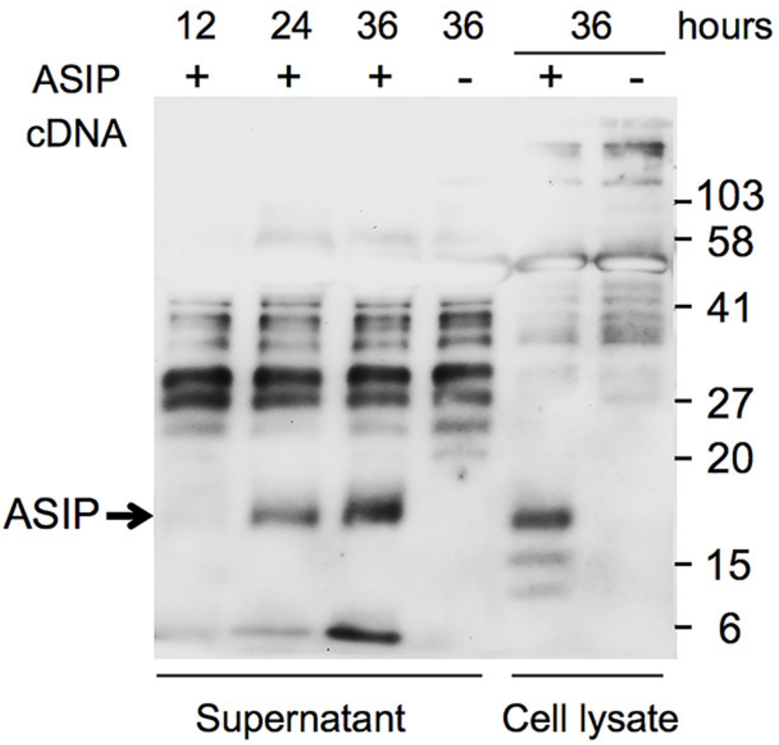

Supplementary Figure S1: Image of entire anti-ASIP Western Blot shown in Figure 1A of cell culture supernatant and cell lysates from HEK293 cells transfected with a mouse ASIP cDNA (as per journal instructions to authors). Lysates of transfected cells show an anti-ASIP reactive band that is absent from untransfected cells and is the approximate size predicted from the amino acid sequence of mature ASIP. Cell-free culture supernatants from these cultures showed a similarly sized anti-ASIP reactive species (indicated by an arrow), as well as non-specific bands that were also present in the supernatant of untransfected cells. The position of molecular size standards are shown on the right.

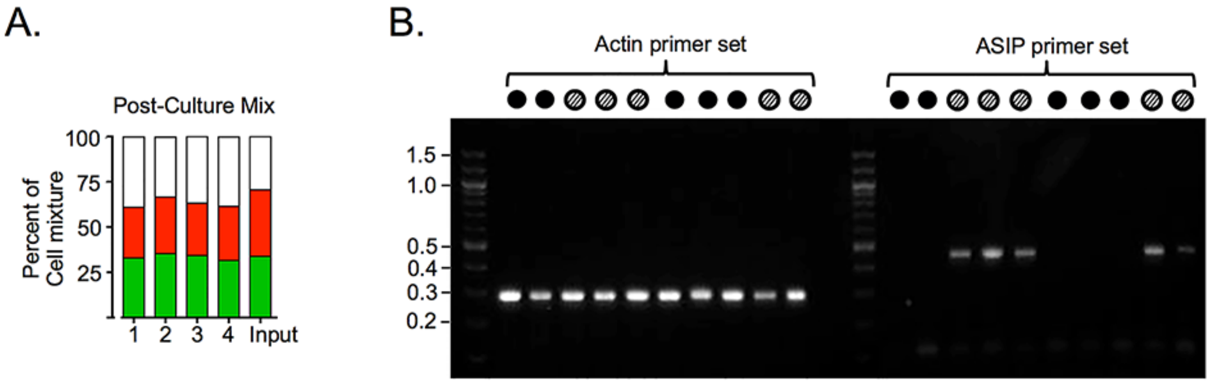

Supplementary Figure S2: A. Confirmation of a lack of growth difference between parental, GFP transduced and ASIP/mChy transduced B16 cells when grown in culture. Replicate separate cultures of parental B16-F10 (white bars), B16-GFP (green bars) and B16-ASIP-mChy (red bars) cells were seeded at low density. Every 24 hr, cells were detached from one plate each, pelleted by low speed centrifugation and resuspended in 1 ml growth medium. Equal volumes of each cell suspension were mixed and the mixture was analyzed by flow cytometry (post-culture mix). **B.** Image of the entire agarose gel after electrophoresis of RT-PCR amplicons as shown in Figure 3A (as per journal instructions to authors). Samples from individual mice in the B16-F10 alone group are indicated by black circles and those from the 1:1 mix group by hashed circles. The position of molecular size standards (in kb) is shown on the left.

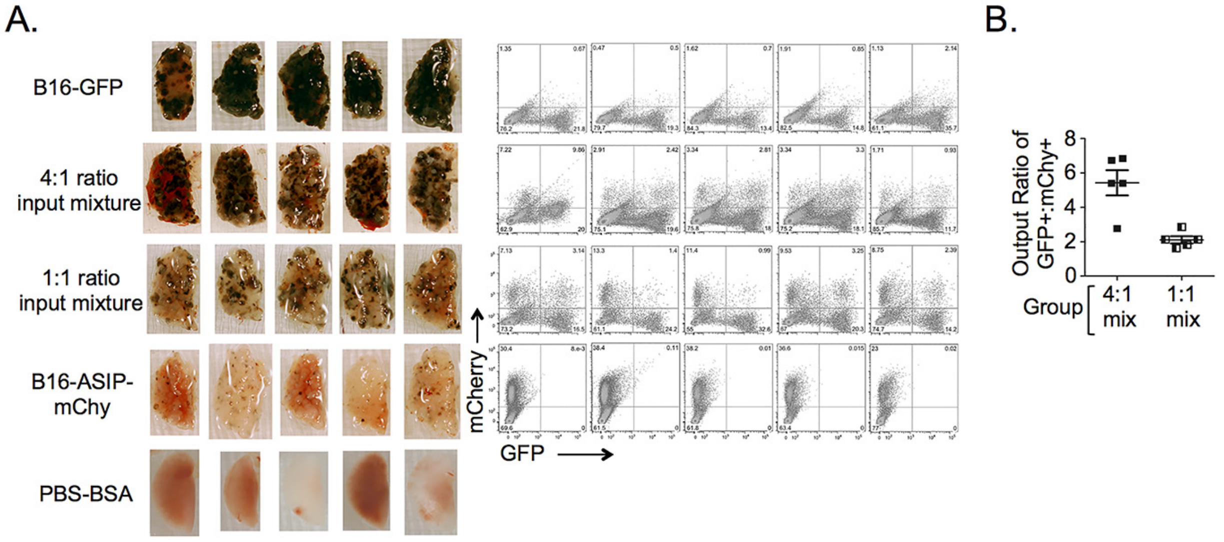

Supplementary Figure S3: **A.** Photographs of the individual fixed lung lobes from each group of mice in the survival experiment presented in Figure 5 are shown on the left. Flow cytometry dot plots of the fresh lung cell suspensions are shown on the right in the same order as the color lung lobe images and the percent of each population is shown in each corner of the individual plots. **B.** Ratios of GFP+ relative to mChy+ tumor cell percentages from total lung populations for individual mice in the 4:1 and 1:1 mix groups (presented as means for the group in Figure 5C) are shown.

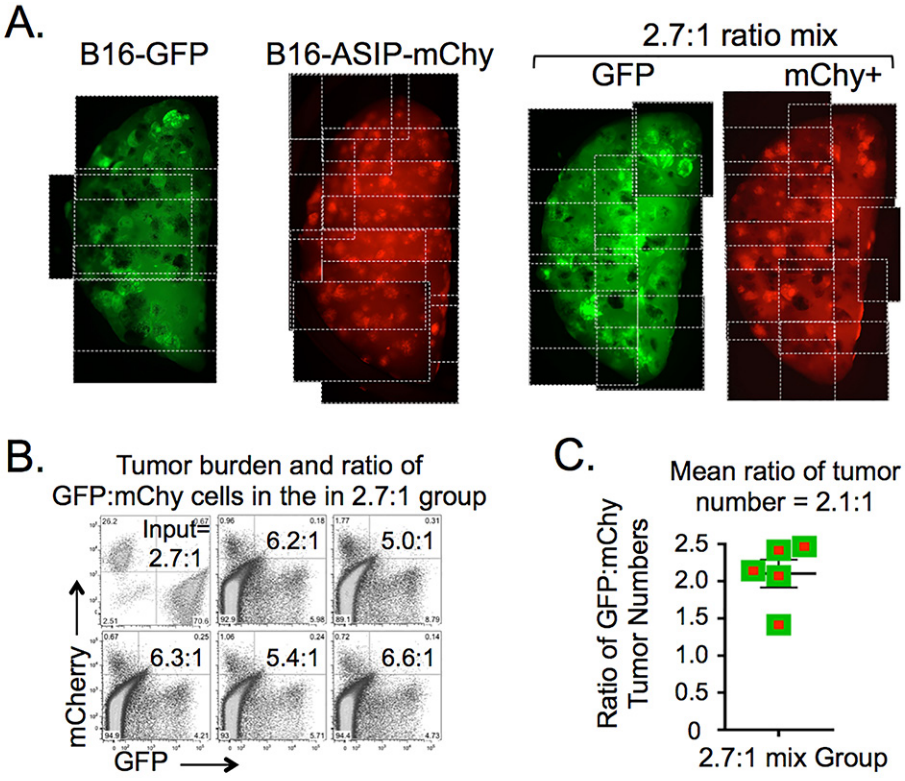

Supplementary Figure S4: **A.** Delineation of individual images used to generate the fluorescent lung composites of fixed lung lobes shown in Figure 7. These images were used to determine tumor foci numbers reported in Figure 6 and to show the general appearance and morphology of tumors as shown in Figure 7B. **B.** Relative tumor burden and ratio of GFP+:mChy+ tumor cells on Day 12 for all mice in the 2.7:1 group of the lung metastasis study shown in Figure 6. **C.** Dotplot of the ratios of GFP+ relative to mChy+ tumor numbers from which the mean ratio reported in Figure 6 was obtained. The ratio from each individual mouse is indicated by a green square with a red center.
